# Miniaturized double-wing ∆E-effect magnetic field sensors

**DOI:** 10.1038/s41598-024-59015-5

**Published:** 2024-05-14

**Authors:** Fatih Ilgaz, Elizaveta Spetzler, Patrick Wiegand, Franz Faupel, Robert Rieger, Jeffrey McCord, Benjamin Spetzler

**Affiliations:** 1 Chair for Multicomponent Materials, Department of Materials Science, Faculty of Engineering, Kiel University, 24143 Kiel, Germany; 2 Nanoscale Magnetic Materials - Magnetic Domains, Department of Materials Science, Faculty of Engineering, Kiel University, 24143 Kiel, Germany; 3 Networked Electronic Systems, Department of Electrical and Information Engineering, Faculty of Engineering, Kiel University, 24143 Kiel, Germany; 4 Micro- and Nanoelectronic Systems, Department of Electrical Engineering and Information Technology, Ilmenau University of Technology, 98693 Ilmenau, Germany

**Keywords:** Sensors and biosensors, Magnetic properties and materials, Magnetic properties and materials, Magnetic devices

## Abstract

Magnetoelastic micro-electromechanical systems (MEMS) are integral elements of sensors, actuators, and other devices utilizing magnetostriction for their functionality. Their sensitivity typically scales with the saturation magnetostriction and inversely with magnetic anisotropy. However, large saturation magnetostriction and small magnetic anisotropy make the magnetoelastic layer highly susceptible to minuscule anisotropic stress. It is inevitably introduced during the release of the mechanical structure during fabrication and severely impairs the device’s reproducibility, performance, and yield. To avoid the transfer of residual stress to the magnetic layer, we use a shadow mask deposition technology. It is combined with a free-free magnetoelectric microresonator design to minimize the influence of magnetic inhomogeneity on device performance. Magnetoelectric resonators are experimentally and theoretically analyzed regarding local stress anisotropy, magnetic anisotropy, and the ΔE-effect sensitivity in several resonance modes. The results demonstrate an exceptionally small device-to-device variation of the resonance frequency < 0.2% with large sensitivities comparable with macroscopic ΔE-effect magnetic field sensors. This development marks a promising step towards highly reproducible magnetoelastic devices and the feasibility of large-scale, integrated arrays.

## Introduction

In today’s technologically advanced world, magnetic field sensors have become essential components across a diverse range of industries^1^, including magnetic recording^2,3^, aerospace^4^, automotive^5^, electronics^6^, and biomedical applications^7–10^. In recent years, thin-film magnetoelectric (ME) sensors have become a class of promising magnetometers for detecting low-frequency and small-amplitude magnetic fields^11–17^. These sensors comprise magnetoelectric composites of mechanically coupled magnetostrictive and piezoelectric components^18,19^. They can be integrated with electronics and have shown potential for array-based applications^20–24^. Magnetoelectric sensors have demonstrated detection limits in the low picotesla regime through the direct magnetoelectric effect; however, their operation is either limited to macroscopic sizes of the sensor elements or high frequencies and narrow bandwidths of a few hertz around the sensor’s resonance frequency^11,17,25^. One way of overcoming these limitations is a modulation technique based on the ΔE effect^26^. The ΔE effect describes the dependency of the mechanical stiffness tensor on the magnetization due to stress-induced magnetostrictive strain^27–32^. Hence, a magnetic field can alter the stiffness tensor of magnetostrictive materials and induce a shift in the resonance frequency^27–32^, which can be read out electrically^26^. ΔE-effect magnetic field sensors designed as plate and cantilever resonators and surface acoustic wave devices have demonstrated detection limits in the sub-nT regime at low frequencies <  < 1 kHz^15,33–39^.

Previous work on cantilever-type ΔE-effect sensors has demonstrated the detrimental influence of inhomogeneous effective magnetic properties around the clamping caused by shape anisotropy and residual stress^32,40,41^. Another persistent challenge connected to residual stress is reproducibility: huge device-to-device performance variations by more than 200% and resonance frequency deviations of 6–10% are reported for magnetoelectric resonators with identical dimensions caused by minimal stress that couples into the magnetic properties via the large magnetostriction^42,43^. Using large effective anisotropies or small magnetostriction reduces the influence of stress on the magnetic properties but simultaneously decreases the sensor’s sensitivity^40,44,45^. Developing a reliable technology for fabricating magnetoelastic sensors is crucial to address these issues. Typically, the samples are fabricated by depositing and structuring the magnetoelastic layer on a constrained resonator, which is subsequently etched out (released)^15,33,37^. During the release process, anisotropic stress is unintentionally introduced into the magnetoelastic layer by the relaxation of intrinsic stress in the substrate and other layers^42^. Consequently, the residual stress in the magnetic layer is not only determined by the magnetic layer deposition process but also by the fabrication of the other underlying layers and the substrate. This results in many process steps and parameters that must be tightly controlled to minimize anisotropic stress. For example, for soft magnetic FeCoSiB and FeGaB layers with saturation magnetostriction ${\lambda }_{{\text{s}}}=20-70~{\text{ppm}}$^46,47^, anisotropic stress of $10-35~\mathrm{MPa}$ is enough to completely reverse the effective magnetic anisotropy (see “Methods” section). Moreover, deposition conditions of the magnetostrictive layers, such as substrate temperature, pressure, and power, have a significant influence on composition^48^, morphology^49,50^, and microstructure^51–53^. Precise control of these parameters is essential as they directly impact the magnetic properties of the films. Solving the reproducibility problem is of utmost importance for large-scale industrial applications and the cost-effective fabrication of magnetoelastic devices. By developing a reliable fabrication technology and minimizing the variation in device performance, the full potential of magnetoelastic resonators can be realized, enabling their widespread adoption in various industries and research fields.

Here, we present a double-wing microresonator design and a deposition technology for fabricating highly reproducible ΔE-effect sensors. Such technology is also relevant for other highly magnetostrictive devices such as pressure sensors^54,55^, actuators^56^, magnetoelectric antennas^57–59^, and magnetic field sensors^42,60–63^. The results represent a significant step toward cheap and reliable device fabrication and large-scale arrays. We analyze the spatial distribution of residual stress and magnetic anisotropy induced during the deposition process following a combined experimental and theoretical approach. The resonators are assessed for the application as ΔE-effect magnetic field sensors in terms of their sensitivity, electromechanical properties, and resonance detuning in several resonance modes. Finally, we explore arrays of parallel-connected sensor elements with identical geometries to investigate reproducibility and compare their performances with single sensor elements.

## Results and discussion

### Resonator design and technology

**Figure 1:**
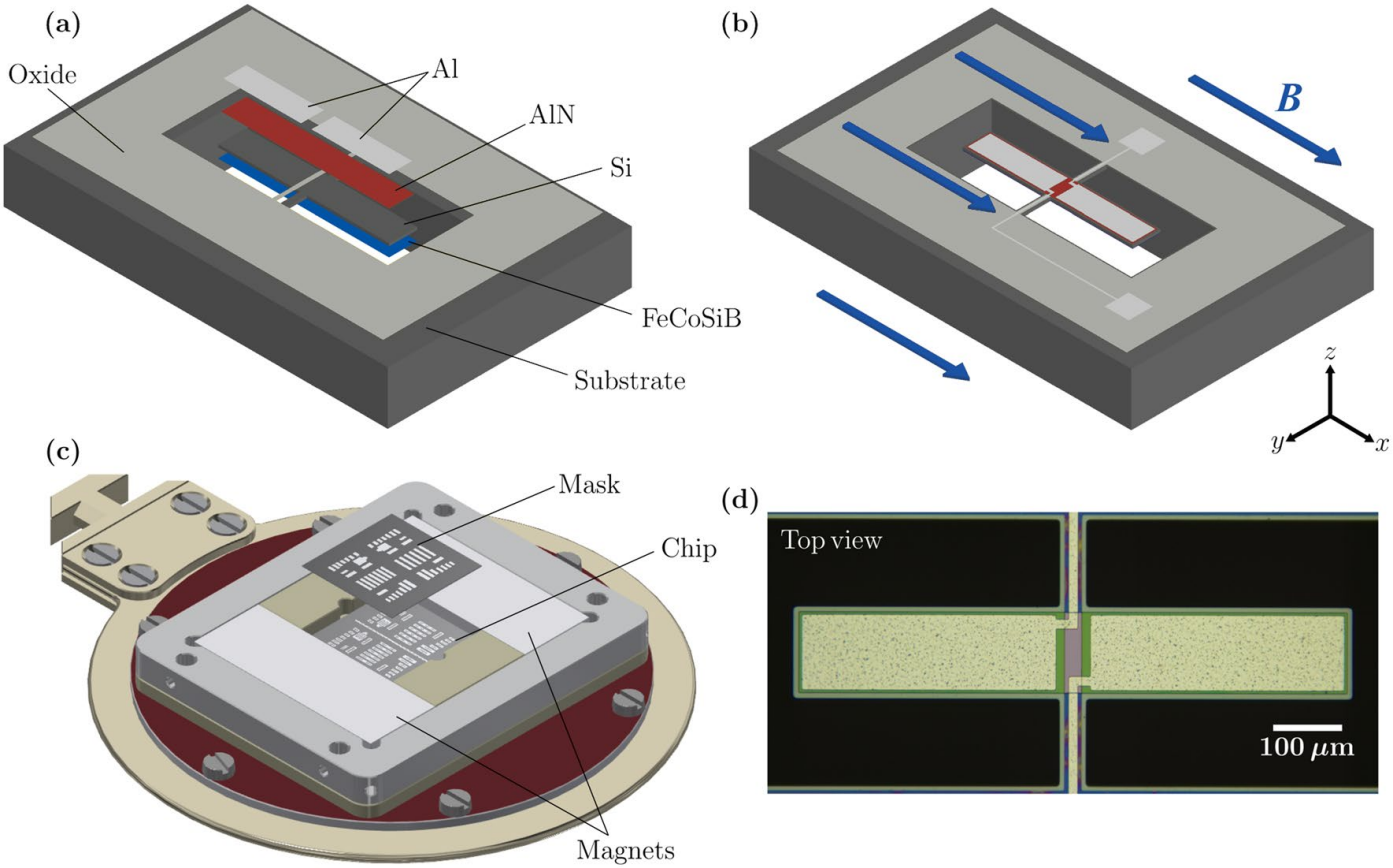
Sensor design and schematic of magnetic layer deposition. (**a**) Schematic of a miniaturized double-wing ΔE-effect sensor with the individual layers. (**b**) Schematic view of the complete sensor during operation with the indicated direction of the applied magnetic field during measurements. (**c**) Illustration of the magnetic layer deposition with shadow-mask deposition technique. (**d**) Optical microscopy image of the top of an example resonator.

The individual resonators (Fig. 1a,b) are based on electromechanical thin-film multilayer structures comprising a 10-µm-thick doped poly-Si substrate, also functioning as a rear-side electrode, with a 0.2-µm-thick pad oxide layer on the top to insulate it electrically from the subsequent layers. On top of the pad oxide layer, a 0.5-µm-thick AlN piezoelectric layer is deposited, followed by two 1-µm-thick patterned Al electrodes symmetrically placed on both sides of the anchors for actuation and read-out. A top view of an example resonator is shown in Fig. 1d. Finally, a 200-nm-thick amorphous magnetostrictive layer (Fe_90_Co_10_)_78_Si_12_B_10_ (FeCoSiB) is deposited on the rear side of the free-standing resonators.

In contrast to typically used lithography methods^33,42,43,64^, the magnetostrictive layer is deposited through a shadow mask onto the rear side of the released resonators using an in-house built magnetron deposition system (sample holder in Fig. 1c). The setup permits micrometer-precision mask alignment for shadow masks with sub-µm feature size. No additional lithography processes for structuring the magnetic layer were used. Because the magnetic layer is structured via the shadow mask and deposited as the last layer on the released resonator, the residual stress in the magnetic layer is only determined by the magnetic layer deposition process and not by the fabrication of the other underlying layers or the substrate. This reduces the degrees of freedom to be controlled during the fabrication and permits precise stress control by adjusting the deposition conditions (see “Methods” section). During the deposition of the magnetic layer, a magnetic field is applied by permanent magnets to induce a magnetic easy axis along the short axis of the resonator.

A second component contributing to the reproducibility and performance is the resonator design. Notable technological advantages result from using double-wing micro-resonators (Fig. 1b) instead of a classical cantilever geometry. Anchoring the double-wing resonator in the center permits homogeneous magnetic layer deposition on the entire resonator. It avoids partial shadowing of the geometry by the substrate, which would occur for cantilevers at the clamping. As we will show in the “Sensitivity and ∆E effect” section, an antisymmetric resonance mode can be excited with the resonator design, which reduces the detrimental influence of magnetic inhomogeneities at the anchor region and the tips. Additionally, the anchor design reduces clamping loss compared to a cantilever design^32,40^ and, thereby, the coupling of adjacent resonator elements via the substrate.

For this work, we fabricated magnetoelastic resonators with various lengths of 400–850 µm and widths of 60–125 µm. The microfabrication and deposition processes are detailed in the “Methods” section. All results presented in the following section are for a representative resonator (ID1 with in-plane dimensions of 640 µm × 105 µm. The data for all other produced samples is available in Supplementary Information.

### Residual stress

**Figure 2:**
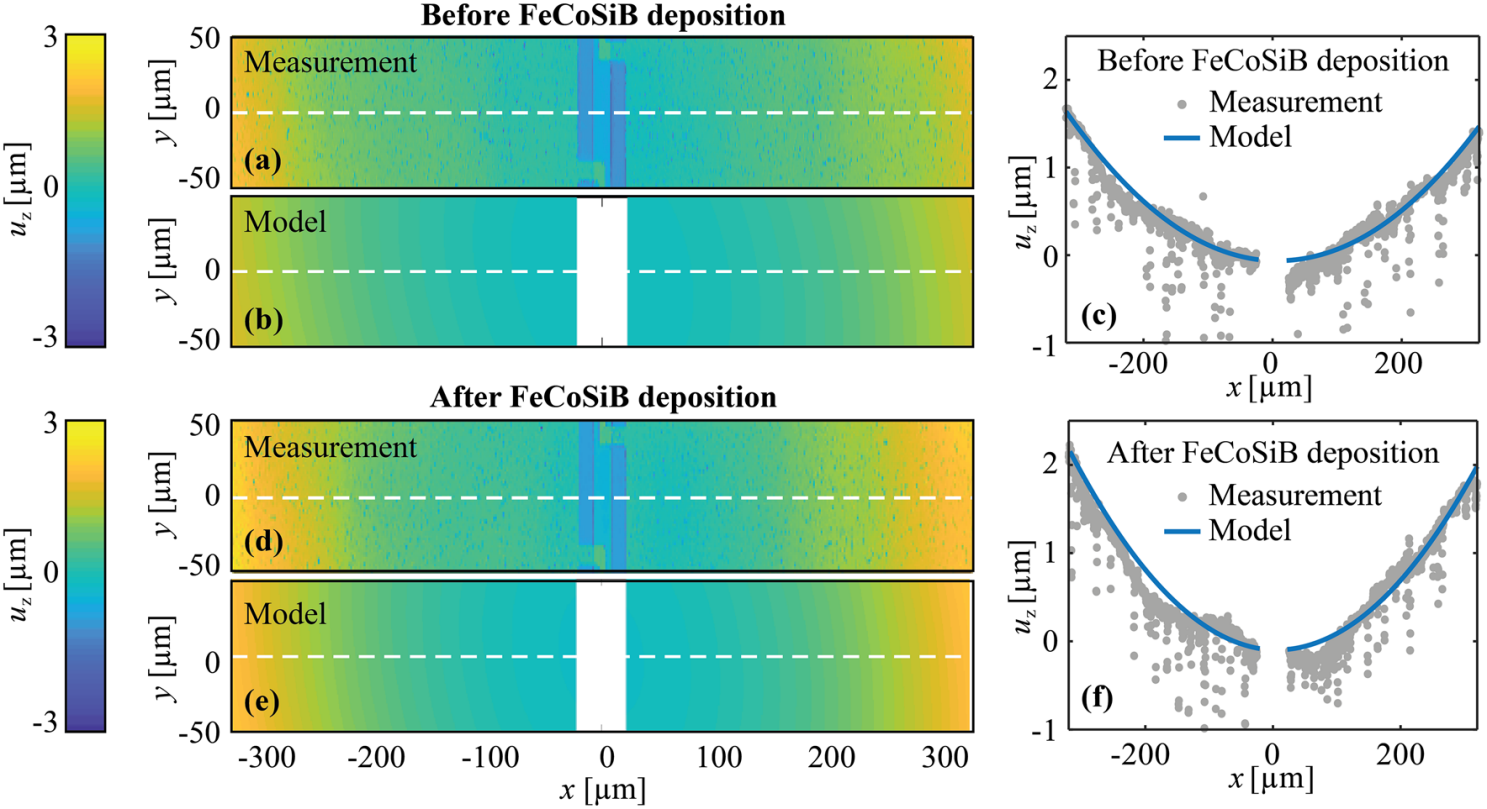
Measured and simulated z-displacement ${u}_{{\text{z}}}$ of a representative sensor. (**a**–**c**) ${u}_{{\text{z}}}$ before and (**d-f**) after FeCoSiB deposition. (**c**) and (**f**) show the data from the cut lines marked with white dashed lines in figures (**a,b**) and (**d,e**), respectively. For the displacement simulation before FeCoSiB deposition (**a**–**c**), an initial isotropic stress of -140 MPa was applied to the substrate, and after the deposition, an additional homogeneous initial stress of ${\sigma }_{11}=-245~\mathrm{MPa}$ and ${\sigma }_{22}=-235~\mathrm{MPa}$ was applied to the magnetic layer.

To demonstrate the stress control of our deposition technology, we analyze the residual stress induced during the deposition of the magnetic layer and its influence on the effective magnetic anisotropy. For that, the out-of-plane displacement ${u}_{{\text{z}}}$ of the representative resonator with in-plane dimensions of 640 µm × 105 µm was measured with a laser profilometer before and after the FeCoSiB deposition. A mechanical finite-element-method (FEM) model is fitted to the measured data to identify intrinsic stress in the substrate and the magnetic layer. Figure 2 shows the measured and simulated ${u}_{{\text{z}}}$ before (Fig. 2a–c) and after (Fig. 2d–f) the FeCoSiB deposition. The scatter in the measured data is caused by noise in the measurements and high roughness of the Al layer where the measurements were conducted. The resonator is slightly bent upwards before the FeCoSiB deposition owing to the relaxation of residual stress in the nonmagnetic layers upon release of the resonator. Simulations and measurements match very well, assuming an isotropic initial stress in the substrate of $-140~\mathrm{MPa}$ (Fig. 2a–c).

After the deposition of the magnetic layer, the displacement of the resonator wings is slightly increased by approximately 0.5 µm at the tips (Fig. 2c,f), indicating deposition-induced compressive stress in the magnetic layer. The simulations and measurements match very well (Fig. 2d–f) for a homogeneous initial stress of ${\sigma }_{11}=-245~\mathrm{MPa}$ and ${\sigma }_{22}=-235~\mathrm{MPa}$ with a minuscule anisotropy of ${\sigma }_{11}-{\sigma }_{22}\approx -10~\mathrm{MPa}$. Because of the shape of the cantilever, the stress anisotropy ${\sigma }_{11}-{\sigma }_{22}$ decreases slightly after the relaxation, reaching an equilibrium value of ${\sigma }_{11}-{\sigma }_{22}\approx 9.3~\mathrm{MPa}$ in the center of the wings (see Supplementary Information). As a result, the stress-induced magnetic anisotropy in the magnetic layer reaches ${K}_{\upsigma }\approx 450~{\text{J}}{\text{m}}^{-3}$ (saturation magnetostriction constant ${\lambda }_{\text{s}}=30\text{ ppm}$^47^) with the magnetic easy axis oriented along the short axis (y-axis) of the resonator.

**Figure 3:**
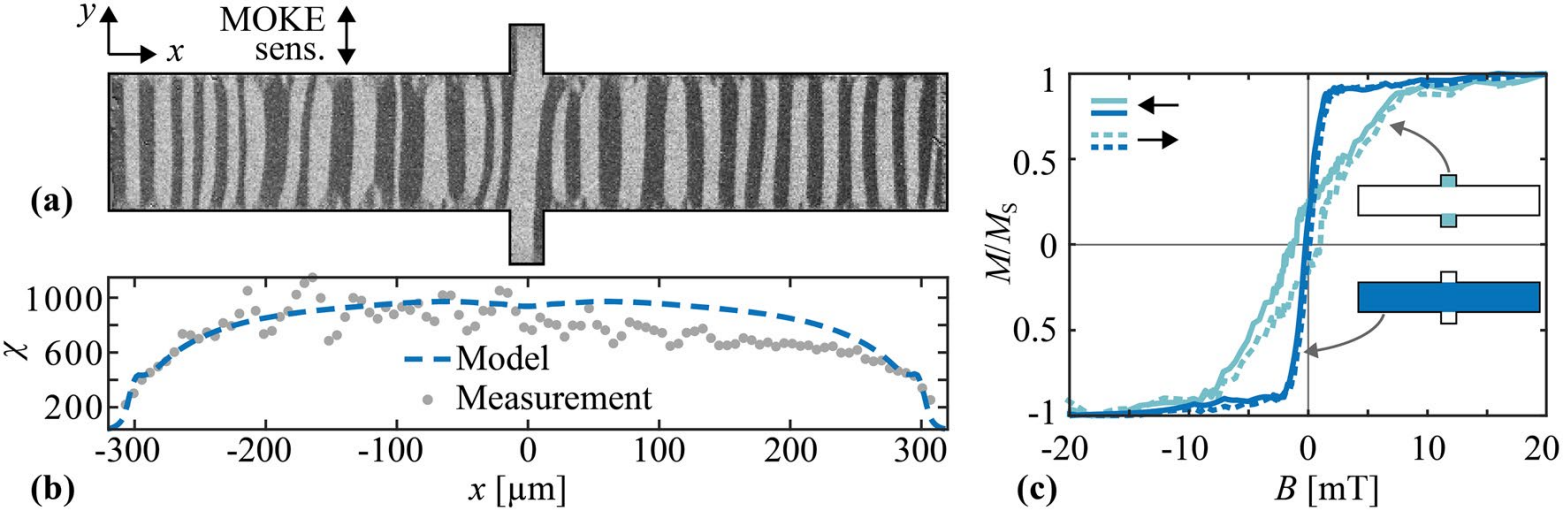
Magnetic properties of the resonator ID1 obtained by magneto-optical Kerr effect (MOKE) microscopy. (**a**) MOKE image of the magnetic domains of the sensor after demagnetizing along the x-axis. The magneto-optical sensitivity axis (MOKE sens.) is aligned with the y-axis as indicated. (**b**) Spatial distribution of the differential magnetic susceptibility χ estimated from the measurements and compared with simulations. (**c**) Local magnetization curves measured in two different regions with $B$ applied along the long axis of the resonator.

To validate the estimation of the stress anisotropies, we employed magneto-optical Kerr effect (MOKE) microscopy^65^. Figure 3a shows the domain configuration of the sample after demagnetization along its long axis. The magnetic domains orient predominantly along the short axis of the cantilever. They bend slightly around the clamping region because of the different stress relaxation at the edges compared to the resonator’s center (see Fig. S1 in Supplementary Information).

We extracted local magnetization curves from additional MOKE measurements to estimate the distribution of the local differential magnetic susceptibility χ at zero field along the sample’s long axis (x-axis). The results are shown in Fig. 3b. In the center of the resonator, the differential susceptibility is $\upchi \approx 950$; it decreases close to the edges to values of $\upchi <400$. To quantify the individual energy contributions that define the value of χ, we use a macrospin model (see “Methods” section and Supplementary Information for details). In the model, we consider magnetoelastic anisotropy energy, demagnetizing field energy, and uniaxial magnetization-induced anisotropy energy introduced during sputtering along the short cantilever axis. FEM simulations are performed to obtain the demagnetizing field energy, while magnetoelastic anisotropy energy density ${K}_{\sigma }$ is taken from the residual stress analysis. The magnetization-induced anisotropy energy density ${K}_{{\text{M}}}$ is considered a fitting parameter. The simulations of $\upchi$ match the measurements (Fig. 3b) for a realistic (and spatially constant) value of ${K}_{{\text{M}}}\approx 500~{\text{J}}{\text{m}}^{-3}$^66^. The simulations also match the decrease of χ at the edges is caused by the demagnetizing field (see Supplementary Information for details). Hence, the residual stress analysis is overall consistent with the measured magnetic properties.

Local magnetization curves were recorded on the beam and anchors to distinguish their local magnetic behavior (Fig. 3c). The magnetization curve recorded on the anchor regions exhibits a significantly different shape than that of the beam region. The reduced slope at $B>0.5~{\text{mT}}$ is mainly caused by the demagnetizing field in the wing regions (see Fig. S2a, Supplementary Information). Similar magnetic properties have been observed for all other resonators produced for this paper, as shown in the Supplementary Information. The inhomogeneity and effective anisotropy of the magnetic properties in the anchor region are expected to deteriorate the sensor performance if this region is active during sensor operation^32,34,36,40^. In the next section, we will show how carefully selecting the resonance mode based on the known distribution of the effective magnetic properties can improve the frequency tunability.

### Resonance modes

**Figure 4:**
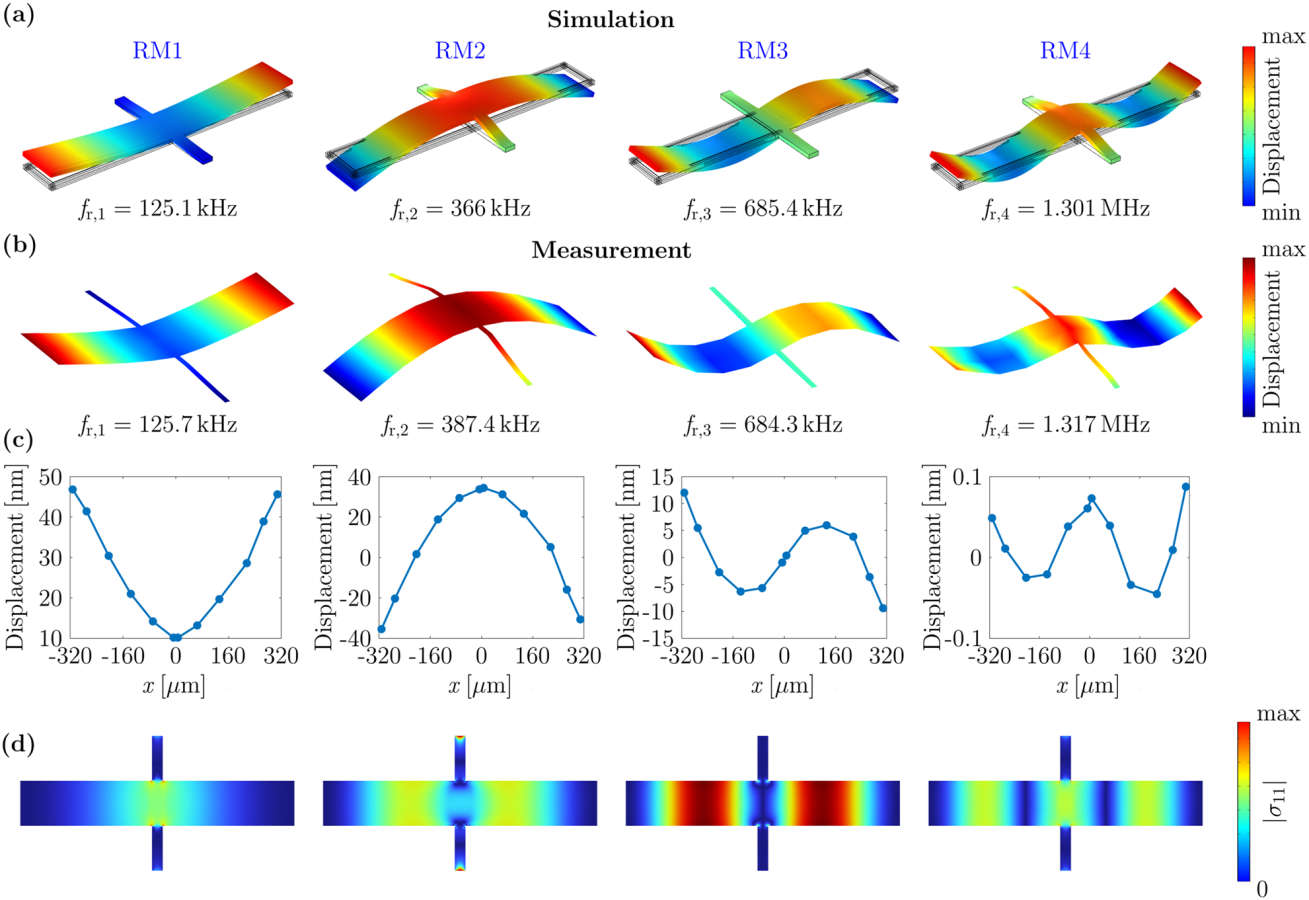
Mode shapes, out-of-displacements and simulated ${\sigma }_{11}$ stress distribution of the first for resonance modes. (**a**) Mode shapes of the first four resonance modes and their corresponding resonance frequencies at the example of sensor ID1 simulated with a FEM model, and (**b**) determined by vibrometer measurements. (**c**) Measured out-of-plane displacements along the center of the long axis of the sensor. (**d**) Distributions of the simulated ${\sigma }_{11}$ component of the stress tensor at the center of the magnetic layer.

To analyze the performance of the exemplary resonator as a ΔE-effect sensor, we selected the first four resonance modes (RM1-4) using FEM simulations and vibrometer measurements. The measured and simulated mode shapes (at $B=0~\mathrm{mT}$) are shown in Fig. 4a–c, with eigenfrequencies of approximately 125.1 kHz (RM1), 366 kHz (RM2), 685.4 kHz (RM3) and 1.3 MHz (RM4). Corresponding frequencies of these modes for other produced resonators are available in Table S1 in Supplementary Information. The simulations match the measurements very well, with minor deviations of the resonance frequencies ${f}_{{\text{r}}}$ smaller than 1.3%, except for RM2, with a deviation of 5.5%. All four modes are of a first or higher-order bending type and differ in their displacement profiles and dynamic stress distributions (Fig. 4d). Among the four resonance modes, only RM3 is asymmetric, with a minimum magnitude of ${\sigma }_{11}$ between the two anchors and a maximum $|{\sigma }_{11}|$ in the center of the resonator wings. In all other modes, it is $\left|{\sigma }_{11}\right|>0$ in the anchor region. The different spatial distribution of the dynamic stress in the resonance modes will allow us to minimize the influence of undesired local magnetic properties by selecting a suitable mode in the next subsection.

### Sensitivity and ∆E effect

One of the main characteristics of a magnetic field sensor is its sensitivity to magnetic fields. As a measure for the sensitivity of the resonator as a ΔE-effect sensor, we use the amplitude sensitivity ${S}_{{\text{am}}} := {S}_{{\text{m}},{\text{r}}}{\cdot S}_{{\text{el}},{\text{r}}}$, as defined previously^32^. It is proportional to the change in resonance frequency ${f}_{{\text{r}}}$ induced by the applied magnetic flux density $B$ via the ΔE effect and to the slope of the sensor admittance $Y(f)$. These two proportionality factors are normalized to the operating frequency and referred to as relative magnetic sensitivity ${S}_{{\text{m}},{\text{r}}}$ and relative electric sensitivity ${S}_{{\text{el}},{\text{r}}}$, respectively. Details are provided in the “Methods” section. In the following, we first analyze the magnetic and electric sensitivities of the different resonance modes and then combine them to draw conclusions about the amplitude sensitivity.

#### Magnetic sensitivity and ∆E effect

**Figure 5:**
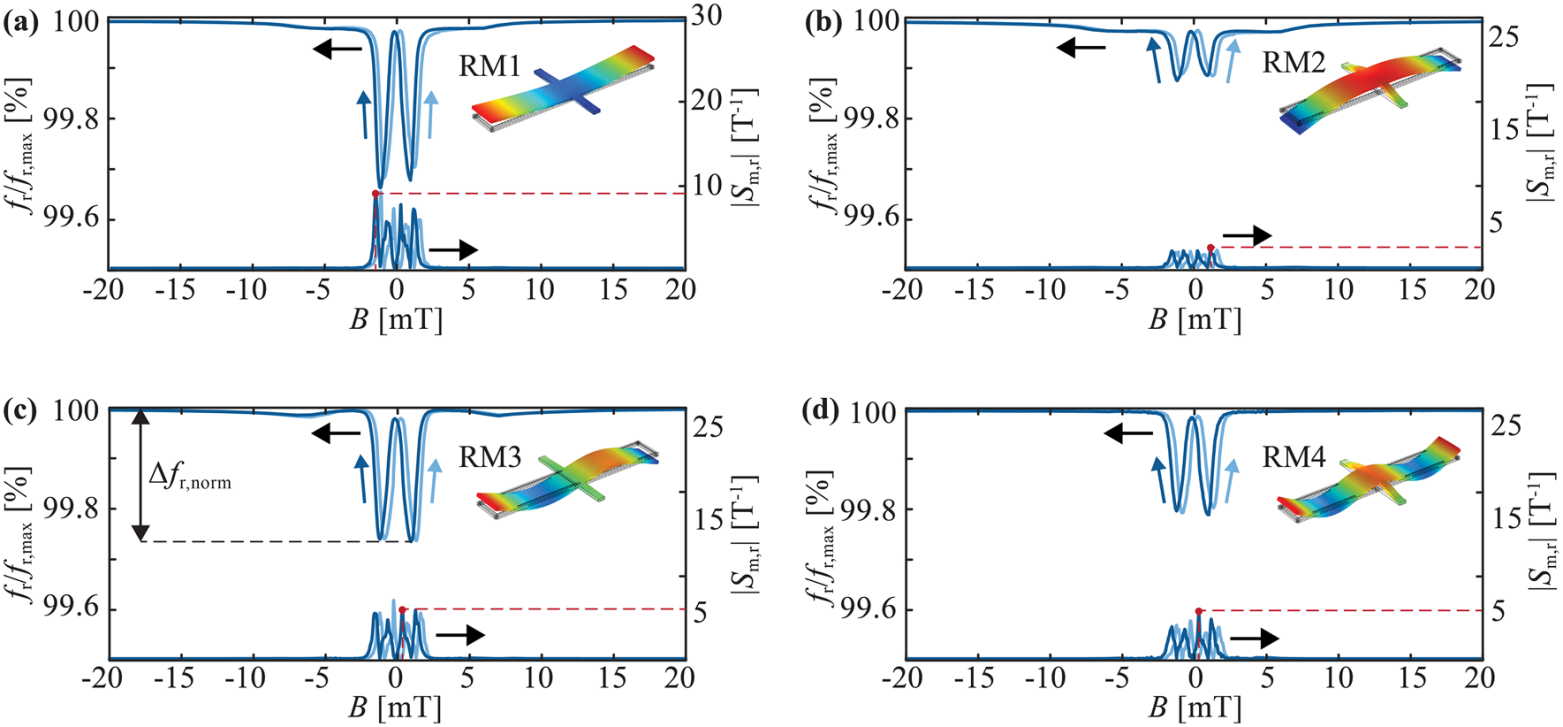
Delta-E effect and magnetic sensitivities. Measured normalized resonance frequencies ${f}_{{\text{r}}}/{f}_{{\text{r}},{\text{max}}}$ and relative magnetic sensitivities ${S}_{{\text{m}},{\text{r}}}$ as functions of the magnetic flux density $B$ applied along the long axis of the resonator for (**a**) RM1 with ${f}_{{\text{r}},{\text{max}}}=125.8$ kHz, (**b**) RM2 with ${f}_{{\text{r}},{\text{max}}}=366$ kHz, (**c**) RM3 with ${f}_{{\text{r}},{\text{max}}}=685.4$ kHz, and (**d**) RM4 with ${f}_{{\text{r}},{\text{max}}}=1302$ kHz. Magnetic working points are indicated with red dots. The black arrows indicate the respective y-axis of the plotted data sets. The dark and light blue arrows indicate the sweep direction of $B$. Dark blue – from 20 mT to − 20 mT, light blue – from -20 mT to 20 mT. The normalized frequency detuning $\Delta {f}_{{\text{r}},{\text{norm}}}$ is indicated with an arrow in (**c**) as an example.

The dependency of the normalized resonance frequency ${f}_{{\text{r}}}(B)/{f}_{{\text{r}},{\text{max}}}$, on the applied magnetic flux density $B$ is shown in Fig. 5 for RM1-4. All four curves are overall w-shaped, typical for bending mode resonators with an effective magnetic anisotropy perpendicular to the main dynamic stress axis and the applied magnetic field^32,33,40^. However, quantitative differences are apparent.

The normalized frequency detuning $\Delta {f}_{{\text{r}},{\text{norm}}} :=({f}_{{\text{r}},{\text{max}}}-{f}_{{\text{r}},{\text{min}}})/{f}_{{\text{r}},{\text{max}}}$, i.e. the difference between the maximum resonance frequency ${f}_{{\text{r}},{\text{max}}}$ and the minimum resonance frequency ${f}_{{\text{r}},{\text{min}}}$ differs significantly between the four resonance modes. It is largest in RM1 with $\Delta {f}_{{\text{r}},{\text{norm}}}$ of 0.33%, followed by RM3 with 0.26% and RM4 with 0.21%. The smallest change of 0.12% is measured in RM2. This leads to correspondingly different relative magnetic sensitivities ${S}_{{\text{H}},{\text{r}}}$, as shown in Fig. 5, with indicated maximum values ${S}_{{\text{m}},{\text{r}}}=8.8~{{\text{T}}}^{-1}$ (RM1), ${S}_{{\text{m}},{\text{r}}}=2.1~{{\text{T}}}^{-1}$ (RM2), ${S}_{{\text{m}},{\text{r}}}=6.0~{{\text{T}}}^{-1}$ (RM3), and ${S}_{{\text{m}},{\text{r}}}=5.5~{{\text{T}}}^{-1}$ (RM4). The same trend is visible for other produced resonators (see Table S2 in Supplementary Information).

The resonance modes also differ in their saturation behavior. RM1 and RM2 show a minuscule increase in the resonance frequency at $\left|B\right|> 5~{\text{mT}}$ before they reach their maximum values, while the resonance frequency in RM3 slightly drops to a local minimum at ≈ 7 mT before increasing again at larger flux densities. These differences in the resonance frequency curves can be well explained by the spatially varying effective magnetic anisotropy. The effective magnetic anisotropy is locally weighted by the alternating stress field of the respective resonance mode, leading to resonance-mode-dependent resonance frequency curves^32^.

The resonance frequency of RM2 (Fig. 5b) is dominated by the elastic properties of the anchors, where the susceptibility of the magnetic layer is comparatively small and highly inhomogeneous (“Residual stress” section). Both factors cause the small frequency detuning of RM2. Consistently, the best performance is obtained in RM1 and RM3, where the contributions of the anchors to the resonance frequency are minor. In RM3, the resonance frequency detuning is slightly smaller than in RM1. This is likely caused by a small contribution of the ${C}_{66}$ stiffness tensor component via the shear stress component ${\sigma }_{12}$ at the anchors (see Supplementary Information), which are twisted during the oscillation in RM3. As demonstrated previously, the superposition of the ∆E effect in the shear component (${C}_{66}$) and the longitudinal component (${C}_{11}$) of the stiffness tensor compensate slightly and can result in an overall reduced frequency detuning compared to a pure bending mode^32^. The contribution of this shear-stress component to the resonance frequency of RM3 explains the different saturation behavior of RM3 compared to the other resonance modes. The slightly increasing resonance frequency at $|B| > 5~\mathrm{mT}$ visible in RM1 and RM2 is caused by the residual nonzero susceptibility visible in the magnetization measurements (“Residual stress” section). In RM3, the increase in resonance frequency is not visible because it is superposed by the contribution of the shear component, which causes the local minimum in the resonance frequency curve at ≈ 7 mT.

We note that there are also other effects, which can cause a dependency of ${f}_{{\text{r}}}$ on the magnetic field, such as magnetostrictive elongation, stress stiffening, and the pole effect^29^. However, previous estimations and simulations have shown that these effects are several orders of magnitude smaller than the ΔE effect in the considered magnetic layers and for typical MEMS resonators^29^.

#### Electric sensitivity and amplitude sensitivity

**Figure 6:**
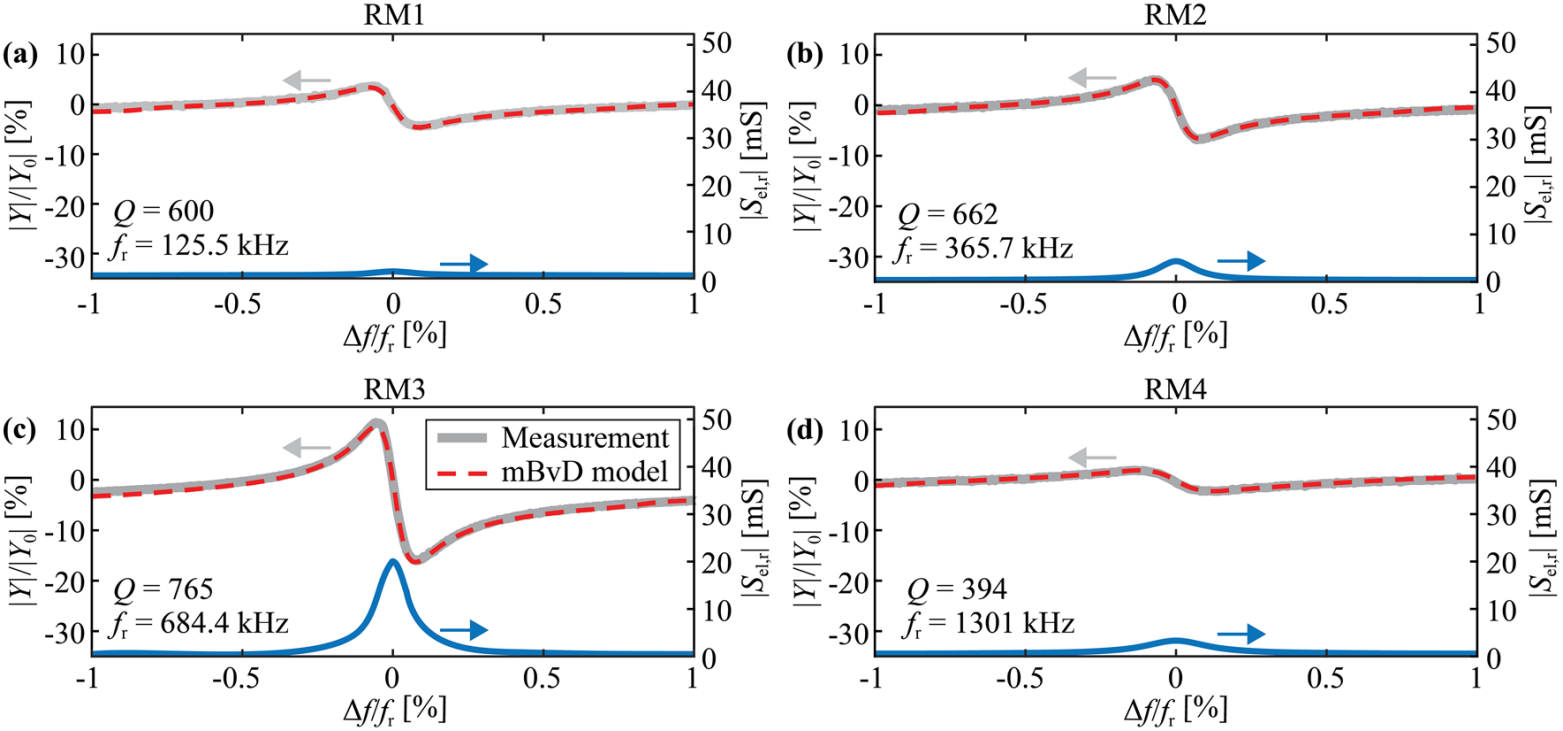
Electrical sensitivities. Measured and modeled normalized admittance magnitude $\left|Y\right|/\left|{Y}_{0}\right|$ and relative electrical sensitivity ${S}_{{\text{el}},{\text{r}}}$ at the magnetic working point as a function of normalized excitation frequency $\Delta f/{f}_{{\text{r}}}$ (with $\Delta f=f-{f}_{{\text{r}}}$) for the first four resonance modes (**a**) RM1 with $\left|{Y}_{0}\right|=8.0~\mathrm{\mu S}$, (**b**) RM2 $\left|{Y}_{0}\right|=23.6~\mathrm{\mu S}$, (**c**) RM3 with $\left|{Y}_{0}\right|=44.8~\mathrm{\mu S}$ and (**d**) RM4 with $\left|{Y}_{0}\right|=82.6~\mathrm{\mu S}$. The resonance frequency ${f}_{{\text{r}}}$ and the quality factor $Q$ at the magnetic working point extracted from the mBvD model are given in the bottom left corners of the subfigures.

Admittance characteristics of RM1-RM4 are shown in Fig. 6a–d as functions of the normalized frequency $\Delta f/{f}_{{\text{r}}}$ with $\Delta f=f-{f}_{{\text{r}}}$ at their respective magnetic working point. The admittance magnitude $\left|Y\right|$ is normalized to its value $\left|{Y}_{0}\right|$ at the resonance frequency ${f}_{{\text{r}}}$ for easier comparison of the data. The relative electric sensitivity is obtained from the derivative of the admittance and plotted in Fig. 6a–d as well. The largest relative electric sensitivity is reached for RM3 with ${S}_{{\text{el}},{\text{r}}}=19.5~{\text{mS}}$. This is approximately a factor of 25 times higher than the maximum electrical sensitivities of RM1, RM2, and RM4, with values of ${S}_{{\text{el}},{\text{r}}}=0.8~{\text{mS}}$, ${S}_{{\text{el}},{\text{r}}}=4~{\text{mS}}$, and ${S}_{{\text{el}},{\text{r}}}=2.8~{\text{mS}}$. A similar trend was observed for most of the other produced resonators (see Table S2 in Supplementary Information). Besides that, resonators with the same geometries except for slightly different anchor widths, e.g. ID 2 and 5, have similar sensitivities, showing that anchors have a minor influence on the sensor performance. High electrical sensitivity in RM3 is expected because the electrodes are specifically optimized for RM3, i.e. they cover the centers of the two wings but only partially cover the center of the resonator between the anchors where the other resonance modes are most active. As a result, the largest amplitude sensitivity is reached in RM3 with ${S}_{{\text{am}}}=121~\mathrm{ \mu S m}{{\text{T}}}^{-1}$. This sensitivity is similar to those previously reported for mm-sized ΔE-effect sensors^32,33,35,36,40,67,68^.

An mBvD model is fitted to the measurements to extract the quality factors of the resonance modes. With values of 535 (RM1), 610 (RM2), 797 (RM3), and 382 (RM4), they are slightly smaller than in previously investigated mm-sized ΔE-effect sensors^36,67,69^. As a consequence, the bandwidth ${f}_{{\text{BW}}}={f}_{{\text{r}}}/Q$ of the microresonator sensors is up to two orders of magnitudes larger due to orders of magnitude higher resonance frequencies. The bandwidths for RM1, RM2, and RM3 are ${f}_{{\text{BW}}}=235~{\text{Hz}}$, ${f}_{{\text{BW}}}=600~{\text{Hz}}$, and ${f}_{{\text{BW}}}=860~{\text{Hz}}$, respectively. RM4 shows the largest bandwidth of ${f}_{{\text{BW}}}=3.4~{\text{kHz}}$ due to its highest ${f}_{{\text{r}}}$ and smallest $Q$ value.

### Sensor arrays

**Figure 7:**
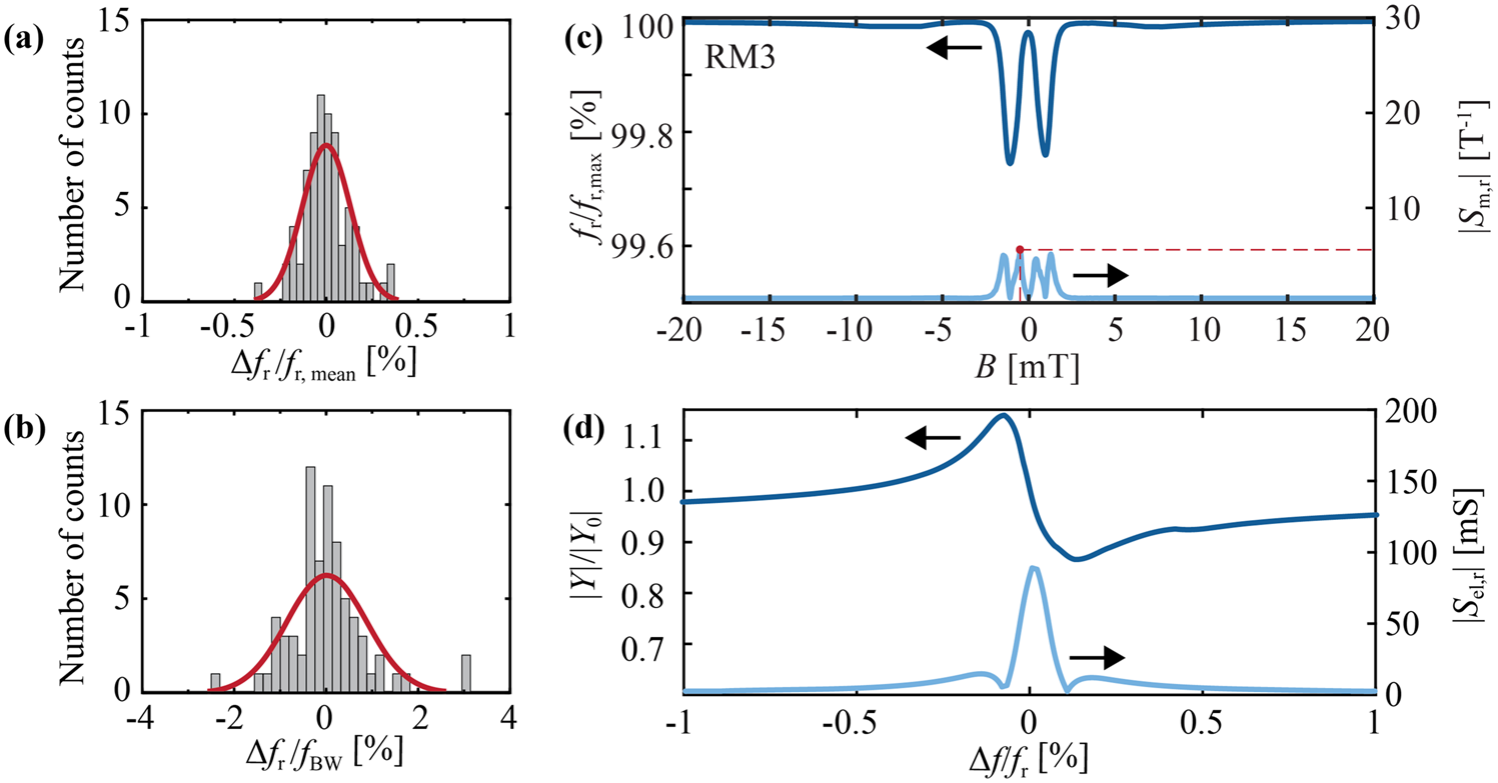
Measured resonance frequency deviations. (**a**) Histogram of all normalized resonance frequency deviations $\Delta {f}_{{\text{r}}}/{f}_{{\text{r}},{\text{mean}}}$, defined via the deviation $\Delta {f}_{{\text{r}}}$ of the resonance frequency ${f}_{{\text{r}}}$ from the mean resonance frequency ${f}_{{\text{r}},{\text{mean}}}$, and (**b**) histogram of the resonance frequency deviations normalized to the averaged resonator bandwidth ${f}_{{\text{BW}}}$ of the resonators for RM1-RM3 in array ID1 (10 resonators) and ID2 (14 resonators). (**c**) Normalized (mean) resonance frequency ${f}_{{\text{r}}}/{f}_{{\text{r}},{\text{max}}}$ with ${f}_{{\text{r}},{\text{max}}}=689~{\text{kHz}}$ and relative magnetic sensitivity ${S}_{{\text{m}},{\text{r}}}$ as a function of the magnetic flux density $B$ applied along the long axis of the resonator for RM3 of array ID1, and (**d**) normalized admittance magnitude $\left|Y\right|/\left|{Y}_{0}\right|$ with ${Y}_{0}=303.8~\mathrm{\mu S}$ and relative electrical sensitivity ${S}_{{\text{el}},{\text{r}}}$ of array ID1 at the working point ($B=- 0.5~{\text{mT}}$) as functions of the frequency of normalized excitation frequency $\Delta f/{f}_{{\text{r}}}$.

To demonstrate the high reproducibility of the resonator characteristics achieved with the presented fabrication method, we examine two arrays of 10 (array ID1) and 14 parallel-connected sensor elements (array ID2). The sensors have in-plane dimensions of 640 µm × 90 µm (array ID1) and 430 µm × 100 µm (array ID2). The resonance frequencies of each sensor in the arrays were measured with a vibrometer. The standard deviation of the resonance frequency variation for the first three resonance modes RM1-3 of 24 sensors is approximately 0.13%, which is 40–50 times smaller than previously investigated magnetoelectric sensors where the magnetic layer was deposited before releasing the resonator^42,43^. In our resonators, RM1 has the highest standard deviation of ≈ 0.16%, followed by RM3 with 0.13% and RM2 with 0.09%. A histogram of the normalized resonance frequency deviation $\Delta {f}_{{\text{r}}}/{f}_{{\text{r}},{\text{mean}}}$ is shown in Fig. 7a. It comprises all resonance frequency data from RM1-3 of both arrays. A histogram of the bandwidth normalized resonance frequency deviation $\Delta {f}_{{\text{r}},{\text{BW}}}$ ($\Delta {f}_{{\text{r}},{\text{BW}}}=\Delta {f}_{{\text{r}}}/{f}_{{\text{BW}}} \approx \Delta {f}_{{\text{r}}}/{f}_{{\text{r}}}\cdot Q)$^67^ is shown in Fig. 7b. Approximately 70% of the resonators have ${f}_{{\text{r}}}$ within the $\Delta {f}_{{\text{r}},{\text{BW}}}<0.5$, which is necessary to improve sensor’s detection limits by noise averaging^67^.

The admittance characteristic of array ID1 with ten parallel connected sensor elements is measured around RM3 at magnetic flux densities from − 20 to 20 mT. Since the resonance frequencies are very similar, we can define an average resonance frequency of the array via an mBvD fit as well. The resulting normalized resonance frequency ${f}_{{\text{r}}}/{f}_{{\text{r}},{\text{max}}}$ is shown in Fig. 5 as a function of the applied magnetic flux density $B$. It follows the same w-shape as the individual sensor element analyzed in the previous sections with a similar minimum of ${f}_{{\text{r}},{\text{min}}}/{f}_{{\text{r}},{\text{max}}}\approx 99.75~\mathrm{\%}$. As expected, also the maximum relative magnetic sensitivity is similar with ${S}_{{\text{m}},{\text{r}}}\approx 4.9~{{\text{T}}}^{-1}$ at $B=-0.5~\mathrm{mT}$ (Fig. 5c and Supplementary Information). The normalized admittance magnitude $\left|Y\right|/|{Y}_{0}|$ and relative electric sensitivity ${S}_{{\text{el}},{\text{r}}}$ at this magnetic operating point are shown in Fig. 7d. With a value of ${S}_{{\text{el}},{\text{r}}}=87.2~\mathrm{mS}$ at ${f}_{{\text{r}}}=688~\mathrm{kHz}$, the relative electrical sensitivity of the array is a factor of 4.5 times larger compared to the single sensor element (“Electric sensitivity and amplitude sensitivity” section, RM3).

The total amplitude sensitivity at the magnetic working point reaches ${S}_{{\text{am}}}=427~\mathrm{\mu S m}{{\text{T}}}^{-1}$, which is approximately 3.5 times larger than with the single sensor element and previously investigated mm-sized ΔE-effect sensors^32,33,35,36^. Overall, the results demonstrate the high reproducibility of the sensors due to excellent stress control with the shadow-mask deposition technology and its potential for sensor arrays.

## Conclusion

We presented a shadow mask deposition technology combined with a free-free magnetoelectric microresonator design for miniaturized ΔE-effect sensors. The deposition of the magnetic layer through the shadow mask avoids residual anisotropic stress from the nonmagnetic layers during microfabrication. Here, we avoid anisotropic stress of estimated up to 30 MPa, which would reduce the magnetic sensitivity by at least a factor of three omitting other detrimental effects such as increase magnetic inhomogeneity and shear stress components (see Supplementary Information). The influence of the magnetic layer deposition on the anisotropic stress and magnetoelastic anisotropy was determined by combining a magneto-mechanical model and (magneto-)optical measurements. A small and homogeneous magnetoelastic anisotropy was achieved ($<500~{{\text{Jm}}}^{-3}$). On the anchors, the demagnetizing field locally increases the effective anisotropy energy density and its inhomogeneity.

The first four resonance modes (RM) were analyzed with FEM simulations and vibrometer measurements. Despite the miniaturization, frequency detuning via the ΔE effect, quality factors, electric sensitivities, and magnetic sensitivities are overall comparable with mm-sized ΔE-effect sensors^32–36,40,67^. Owing to the miniaturized design, larger resonance frequencies between 125.1 kHz and 1.3 MHz are achieved, resulting in significantly higher resonator bandwidths from 0.2 to 3.4 kHz.

The asymmetric bending mode RM3 was identified as a particularly suitable resonance mode for device operation. It avoids the unfavorable inhomogeneous magnetic layer properties on the resonator anchors, can be well excited electrically, and its large resonance frequency results in a higher resonator bandwidth than RM1. In RM3, a maximum amplitude sensitivity of ${S}_{{\text{am}}}=121~\mathrm{\mu S}$ was measured with a bandwidth of 860 Hz, a relative electric sensitivity of ${S}_{{\text{el}},{\text{r}}}=19.5~\mathrm{mS},$ and a relative magnetic sensitivity of ${S}_{{\text{m}},{\text{r}}}=6~{{\text{T}}}^{-1}$.

Arrays of parallel-connected magnetoelectric resonators demonstrated exceptional reproducibility with standard deviations < 0.2% of resonance frequencies. This is 40–50 times smaller than previously investigated magnetoelectric sensors^42,43^. Using arrays of parallel connected sensor elements, the electric and total amplitude sensitivities were improved significantly by a factor of 4.5 and 3.5, respectively, compared to single sensor elements. Combined with the resonance modes we identified, the microresonator design offers multiple benefits compared to traditional cantilevers and marks a notable step toward miniaturized ΔE-effect sensor arrays. The results demonstrate promising progress in the fabrication technology for highly reproducible magnetoelastic structures and devices.

## Methods

### Device fabrication

The magnetoelectric MEMS resonators were designed at Kiel University and fabricated at MEMSCAP Inc. by using a 5-mask level silicon-on-insulator (SOI) patterning and etching process. The magnetostrictive layers were deposited in a final step at Kiel University. The fabrication of the resonators starts from a 150-mm-wide double-side polished (100)-oriented SOI wafer. The SOI wafer comprises a 400-µm-thick substrate, 10-µm-thick polysilicon, and 1-µm-thick oxide layer. The polysilicon layer is doped to serve as the bottom electrode. It is patterned and etched down to the oxide layer. An 0.2-µm-thick thermal oxide layer is grown and patterned to isolate the doped polysilicon layer from the following layers electrically. An 0.5-µm-thick piezoelectric AlN layer is deposited by reactive sputtering and is then patterned and wet etched. After the deposition of the AlN layer, a stack of 20-nm-thick Cr and 1-µm-thick Al is deposited and patterned through a liftoff process. Polysilicon is patterned and etched by deep reactive ion etching (DRIE) down to the oxide layer. A polyimide coat is applied to the top surface of the patterned polysilicon layer to keep the wafer together during the trench etching. After the reversal of the wafer, the bottom side of the substrate is patterned and etched to the bottom side oxide layer by reactive ion etching (RIE). The substrate layer is etched to the oxide layer by DRIE. The oxide layer in the area of the defined trench is removed by wet etching, and finally, the protective polyimide is removed by dry etching. Further details about the microfabrication process can be found elsewhere^70^.

### Estimation of critical stress

For estimating the critical stress that can reverse the effective anisotropy, we assume an initial effective uniaxial anisotropy energy density of ${K}_{{\text{eff}}}=1~{\text{kJ}}/{{\text{m}}}^{3}$ and consider a uniaxial magnetoelastic anisotropy with energy density ${K}_{\sigma }=3{\lambda }_{{\text{s}}}\sigma /2={K}_{{\text{eff}}}$ perpendicular to the initial effective anisotropy axis. Using saturation magnetostriction values between ${\lambda }_{{\text{s}}}=20-70~{\text{ppm}}$^46,47^, yields a critical stress of $10-35$ MPa.

### Magnetic layer deposition

FeCoSiB with a thickness of 200 nm was deposited on the released resonators from their rear side with 10 nm Ta adhesion and capping layers. A shadow mask of the same size as the chip was placed directly on the rear side of the chip. The deposition was carried out at a working pressure of $3\times {10}^{-3}~\mathrm{mbar}$ with an Ar gas flow of 38 sccm and a power of 20 W. The deposition conditions were chosen to achieve the minimum stress arising from the deposition process. During the deposition, a magnetic field of 130 mT was applied by using two Nd_2_Fe_14_B permanent magnets (Fig. 1c) with dimensions of 22 mm × 8 mm × 3 mm to induce a uniaxial magnetic anisotropy perpendicular to the long axis of the resonators. After placing the chip and the mask, the magnets and the frame are covered by a top cover to avoid magnetic layer deposition on the sample holder and the magnets.

### Magnetic characterization

Magnetic properties of the sensors were analyzed using magneto-optical Kerr effect (MOKE) microscopy^65^. To illustrate the domain configuration of the sample in the ground state (Fig. 3a), the sample was demagnetized in a decaying sinusoidal magnetic field applied along its long axis (x-axis). The magneto-optical sensitivity axis was aligned along the short axis of the resonator (y-axis). The quasistatic magnetization curves in Fig. 3c were recorded with the external magnetic field and the sensitivity axis oriented along the x-axis. The distribution of the differential magnetic susceptibility shown in Fig. 3b was estimated from local MOKE magnetization curves like the ones shown in Fig. 3c. The data are then compared with a macrospin magnetization model. The model considers magnetoelastic anisotropy energy, demagnetizing field energy, uniaxial magnetization-induced anisotropy energy, and Zeeman energy^40,71^. Local magnetization curves are then calculated by minimizing the energy of the macrospin considering local values of the stress-induced anisotropy and the demagnetizing field. A saturation magnetization of ${M}_{{\text{s}}}= 1.5~\mathrm{T}$ was used for the simulation^47^. A more detailed description of the model is provided in the section S4 of the Supplementary Information.

### Vibrometer measurements

Vibrometer measurements were performed using a Polytec MSA-500 Micro System Analyzer to determine resonance mode shapes and out-of-plane displacements. The sensor was electrically excited with a sinusoidal voltage with an amplitude of 100 mV via one top electrode at $B = 0~\mathrm{mT}$. The top layer of the sensor was scanned with a focused laser with a 10-times objective lens using a grid including 57 data points in total. Data points were selected on the Al electrodes and the conduction lines.

### Definition of the sensitivities

As a measure for the sensitivity, we use the amplitude sensitivity, as defined previously^32^,

1 $${S}_{{\text{am}}} := {\left.\frac{\partial \left|Y\right|}{\partial B}\right|}_{B={B}_{0}, f={f}_{{\text{r}}}}={S}_{{\text{m}},{\text{r}}}{\cdot S}_{{\text{el}},{\text{r}}},$$

with the electric sensor admittance $Y$ relative magnetic sensitivity ${S}_{{\text{m}},{\text{r}}}$ and the electric sensitivity ${S}_{{\text{el}},{\text{r}}}$,

2 $${S}_{{\text{m}},{\text{r}}}=\frac{1}{{f}_{{\text{r}}}}{\left.\frac{\partial {f}_{{\text{r}}}}{\partial B}\right|}_{B={B}_{0}} , { S}_{{\text{el}},{\text{r}}}={\left.\frac{\partial \left|Y\right|}{\partial f}\right|}_{f={f}_{{\text{r}}}}\cdot {f}_{{\text{r}}},$$

with the resonance frequency ${f}_{{\text{r}}}$, the magnetic bias flux density $B={B}_{0}$ for an operating frequency $f={f}_{{\text{r}}}$.

### Resonance frequency detuning

To determine the resonance frequency as a function of the magnetic bias flux density $B$ shown in Figs. 5 and 7, we measured the admittance magnitude as a function of excitation frequency ${f}_{{\text{ex}}}$ for the magnetic flux density applied along the long axis of the sensor starting from -20 mT to 20 mT and back. Admittance measurements were carried out with an Agilent 4294A Precision Impedance Analyzer. The resonators were excited via one of the top electrodes with an excitation amplitude of 50 mV. We selected ${u}_{{\text{ex}}}=50~\mathrm{mV}$ because resonator nonlinearities set in at large excitation voltage, which leads to a reduction in electrical sensitivity (see Supplementary Information). The resonance frequencies ${f}_{{\text{r}}}$ and quality factors $Q$ are obtained by fitting a modified Butterworth-van-Dyke (mBvD) model to the admittance measurements^72^.

### Finite element method simulations

All finite element simulations were performed in COMSOL Multiphysics^®^ v. 6.0^73^ with the material parameters and layer thicknesses provided in Table 1. The in-plane dimensions and anchor geometry are provided in Table S1 (Supplementary Information, Sensor ID1). The model geometry comprises all layers except for the negligible thin oxide layer and Cr spacers in the electrodes. For all mechanical simulations, we solve the linear mechanical equations of motion with fixed boundary conditions for the displacement at the anchors.

**Table 1:** Model parameters.

Material	${E}_{{\text{m}}}~$[GPa]	$\nu$	$\rho~$[${{\text{kgm}}}^{-3}$]	Thickness
Poly-Si	160	0.22	2300	10 µm
FeCoSiB	150	0.3	7600	200 nm
AlN	330	0.24	3300	0.5 µm
Al	70	0.33	2700	1 µm

Young’s modulus ${E}_{m}$ in magnetic saturation, Poisson’s ratio $\nu$, density $\rho$, and thickness of the layers used in the FEM models.

The resonance modes (“Resonance modes” section) were calculated with an eigenfrequency study using isotropic damping obtained from the measured quality factors.

For the residual stress analysis (“Residual stress” section), the equilibrium stress $\sigma ={\sigma }_{0}+C:\left(\varepsilon -{\varepsilon }_{0}\right)$ and the equilibrium strain $\varepsilon$ were calculated by solving the mechanical equation of motion for static equilibrium conditions. For the simulations, we used ${\sigma }_{0}$ as a fitting parameter and set the initial strain ${\varepsilon }_{0}$ to zero. First, a model geometry without a magnetic layer was used to fit the out-of-plane displacement ${u}_{{\text{z}}}$ to the measurements of the released resonator without a magnetic layer. Here, the initial stress was applied to the Si substrate. Then, in a second model, the magnetic layer was added to the model geometry, and initial stress ${\sigma }_{0}$ is applied to the FeCoSiB layer. The resulting ${u}_{{\text{z}}}$ of the sample is obtained by summing up the displacement fields from both simulations, which is justified by the linearity of the underlying equations.

The local demagnetizing field ${H}_{{\text{D}}}$ was calculated by solving the magnetostatic equations considering the magnetization of the whole sample aligned along the x-axis. The magnitude of the magnetization is equal to the saturation magnetization ${M}_{{\text{s}}}$. For all simulations, we consider ${M}_{{\text{s}}}= 1.5~\mathrm{ T}$^47^. The demagnetizing field energy can then be expressed as ${U}_{D}=-0.5{\mu }_{0}{H}_{{\text{D}},x}{M}_{{\text{S}}}{m}_{x}$, where ${\mu }_{0}$ is the magnetic permeability of vacuum, ${H}_{{\text{D}},x}$ is the local x-axis component of the demagnetizing field. Here, we only consider the demagnetizing field along the x-axis to approximate the flux closure owing to domain formation.

### Supplementary Information

Supplementary Information.

## Data Availability

The data presented in this study are available on request from the corresponding author.
