# Supplementary material for: Miniaturized double-wing ∆E-effect magnetic field sensors
Source: Sci Rep. 2024 May 14;14:11075. doi: 10.1038/s41598-024-59015-5 (PMC11094197; doi:10.1038/s41598-024-59015-5)
Supplement: Supplementary file 1 — Supplementary Information. [file 41598_2024_59015_MOESM1_ESM.pdf]

Supplementary Information

Miniaturized Double-Wing ΔE-Effect Magnetic Field Sensors

Fatih Ilgaz[1], Elizaveta Spetzler[2], Patrick Wiegand[3], Franz Faupel[1], Robert Rieger[3], Jeffrey McCord[2], Benjamin Spetzler[4,*]

[1]Chair for Multicomponent Materials, Department of Materials Science, Faculty of Engineering, Kiel University, 24143 Kiel, Germany

[2]Nanoscale Magnetic Materials - Magnetic Domains, Department of Materials Science, Faculty of Engineering, Kiel University, 24143 Kiel, Germany

[3]Networked Electronic Systems, Department of Electrical and Information Engineering, Faculty of Engineering, Kiel University, 24143 Kiel, Germany

[4]Micro- and Nanoelectronic Systems, Department of Electrical Engineering and Information Technology, Ilmenau University of Technology, 98693 Ilmenau, Germany

*Correspondence: benjamin.spetzler@tu-ilmenau.de

S1. Equilibrium Stress in the Magnetic Layer After Deposition

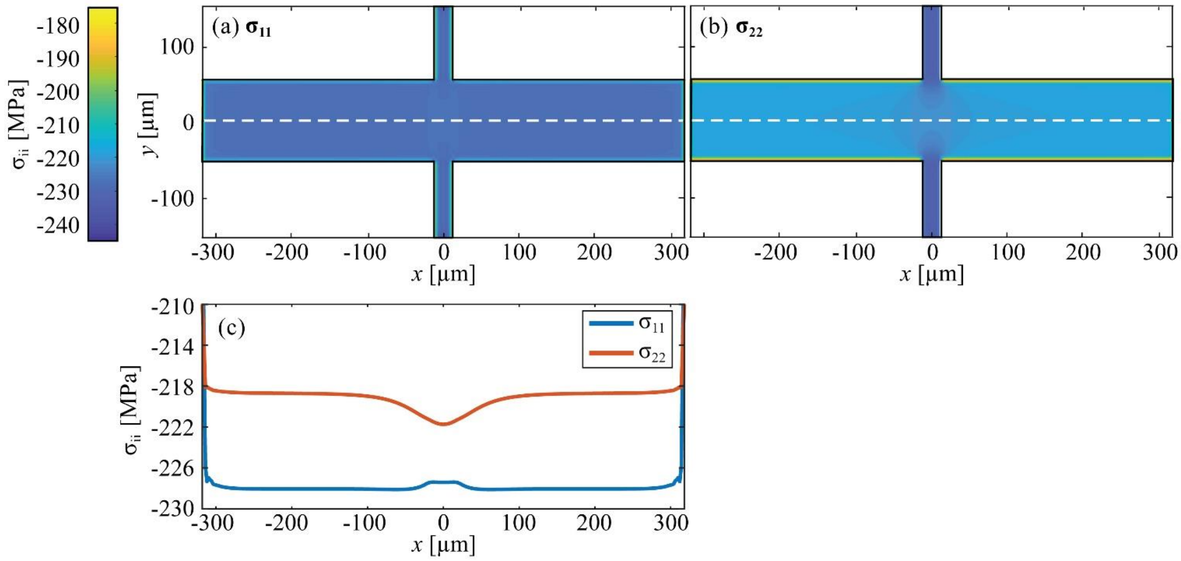

Figure S1. Spatial distribution of the equilibrium stress components in the center plane of the magnetic layer simulated with the FEM model. Initial stress $\sigma_{11} = -245$ MPa and $\sigma_{22} = -235$ MPa was applied to the whole volume of the magnetic layer. (**a**)-(**b**) Spatial distribution of the equilibrium stress components σ_{11} and σ_{22}. (**c**) Distribution of the equilibrium σ_{11} and σ_{22} along the cut line marked with the white dashed line in (**a**) and (**b**).

S2. Spatial Distribution of the Magnetic Anisotropy

The simulated demagnetizing field is shown in Figure S1a,b, and the stress anisotropy $\sigma_{11} - \sigma_{22}$ after relaxation in the center of the magnetic layer in Figure S1c,d.

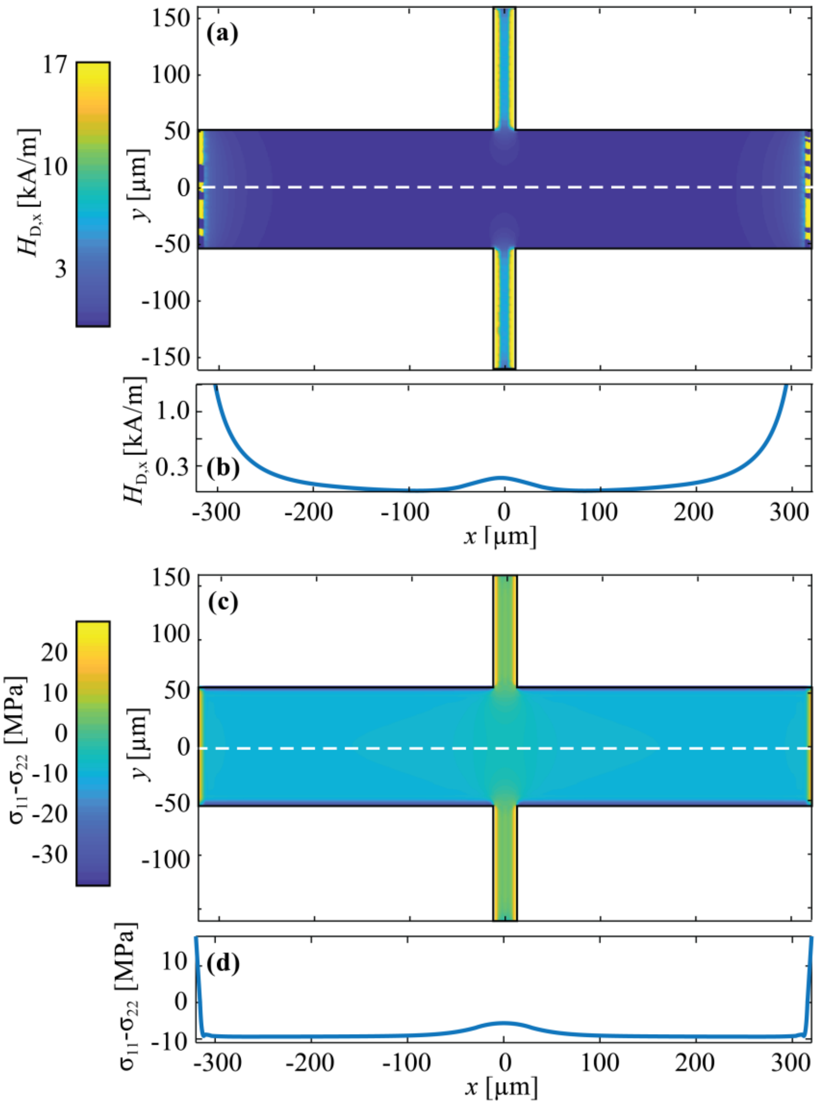

Figure S2. Simulated with FEM models spatial distribution of magnetic properties. (**a**)-(**b**) Distribution of the x-axis component of the demagnetizing field $H_{D,x}$ for the magnetization fixed along the x-axis pointing to the left. (**c**)-(**d**) Anisotropy $\sigma_{11} - \sigma_{22}$ of the in-plane stress in the magnetic layer after deposition in equilibrium. (**b**) and (**d**) show a cut line through the plots in (**a**) and (**c**), respectively.

S3. Initial Stress Anisotropy in the Substrate

Sensor ID 4, which has the same dimensions with only 5 µm difference in anchor width compared to sensor ID 1 (analyzed in detail in the manuscript), was investigated as an example to estimate the anisotropic stress that would be induced in the magnetic layer if it was deposited before the release of the resonator. Displacement measurements prior to magnetic layer

deposition are compared to simulations of different initial stress combinations in the substrate. Figure S3 shows the root mean square deviations (RMSD), between the measured and simulated out-of-plane displacement data of sensor ID 4 for various initial stresses in the substrate. The white dashed line approximately indicates an uncertainty regime of and RMSD of 0.1 µm, which corresponds to 5 % of the maximum deflection of 2 µm observed for this resonator. In addition to the isotropic initial stress of -140 MPa for σ_{11} and σ_{22} as observed in sensor ID 1, the measured and simulated data matches well for various anisotropic stress values up to $|\sigma_{11} - \sigma_{22}| \approx 30$ MPa and various shear stress components σ_{12}. Within the range of $\sigma_{12} = 0$ to $\sigma_{12} = -10\,\text{MPa}$ the shear stress had no significant influence on the RMSD between measured and simulated displacements. This is expected because the comparatively small width of the resonators results in a large uncertainty for bending around the x-axis.

Within a best-case estimation (i.e., omitting shear stress and additional inhomogeneities), $|\sigma_{11} - \sigma_{22}| \approx 30$ MPa would result in an additional magnetoelastic anisotropy contribution of approximately $3\lambda_s\sigma/2 \approx 1350\ \text{J/m}^3$ (with $\lambda_s = 30\,\text{ppm}$), which would decrease the magnetic susceptibility in the center by approximately a factor of two, and correspondingly decrease the magnetic sensitivity $S_m \propto \chi^{3/2}$ [1] by a factor of approximately three.

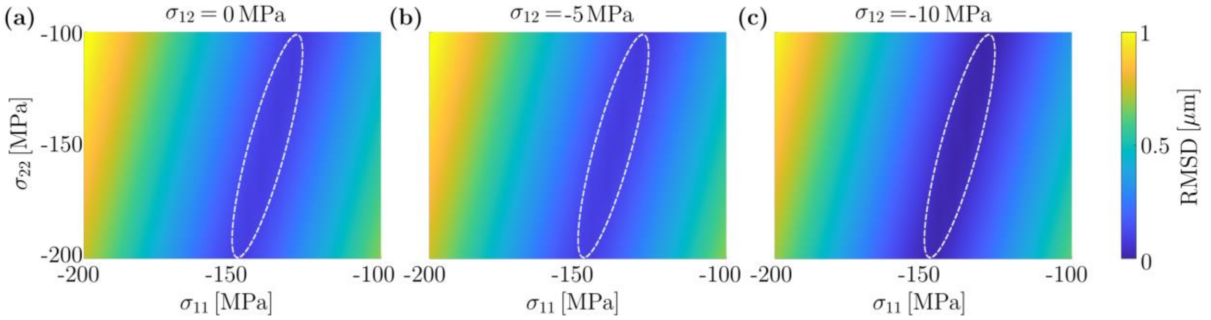

Figure S3. Root mean square deviations (RMSD) between the measured and simulated out-of-plane displacement data of sensor ID 4 for initial stresses ranging from -100 MPa to -200 MPa for σ_{11} and σ_{22}, and (**a**) 0 MPa, (**b**) -5 MPa, and (**c**) -10 MPa for σ_{12} in the substrate before the magnetic layer deposition. Regions inside the dashed ellipses indicate stress values yield less than 0.1 µm deviation (corresponds to approximately 5% relative to the maximum deflection of 2 µm) between the measured and simulated displacement data.

S4. Macrospin Model

The macrospin model used in the paper considers magnetoelastic anisotropy energy density U_σ, demagnetizing field energy density U_D, uniaxial magnetization-induced anisotropy energy density U_M, and Zeeman energy density U_Z[1,2]. Local magnetization curves are calculated by

minimizing the total energy density U of the macrospin for varying values of the magnetic field H applied along the x-axis;

$$
\begin{gathered}
U = U_\sigma + U_\mathrm{D} + U_\mathrm{M} + U_\mathrm{Z} \\
U_\sigma = -\frac{3}{2}\lambda_S[\sigma_{11}\left((\cos\varphi)^2 - \frac{1}{3}\right) + \sigma_{22}\left((\sin\varphi)^2 - \frac{1}{3}\right)] \\
U_\mathrm{D} = \frac{1}{2}\mu_0 M_\mathrm{S} H_{D,x}(\cos\varphi)^2 \\
U_\mathrm{M} = K_\mathrm{M} - K_\mathrm{M}(\sin\varphi)^2 \\
U_\mathrm{Z} = -\mu_0 M_\mathrm{S} H\cos\varphi
\end{gathered}
\qquad \text{(S1)}
$$

Here, $\lambda_S = 35$ ppm[3,4] – saturation magnetostriction, σ_{ij} – components of the stress tensor obtained with the FEM model (Figure S1), φ – magnetization angle, $H_{\mathrm{D,x}}$ - demagnetizing field obtained with the FEM model (Figure S2a-b), $K_\mathrm{M} = 500$ Jm^{-3} – magnetization-induced anisotropy energy density.

Differential magnetic susceptibility χ (Figure S4a and Figure S4b) was calculated from the obtained magnetization curves by taking a slope of the linear fit around $H = 0$ A/m.

Figure S4 shows individual energy contributions to the total energy of the macrospin at three different positions on the x-axis. One can see that demagnetizing field energy U_D plays a major role close to the edges (Figure S4b), and stress-induced anisotropy energy U_σ becomes dominant in the center of the wings and the resonator (Figure S4c-d). A slight increase of U_D is noticeable in the center of the resonator (Figure S4c) due to the shape of the anchors.

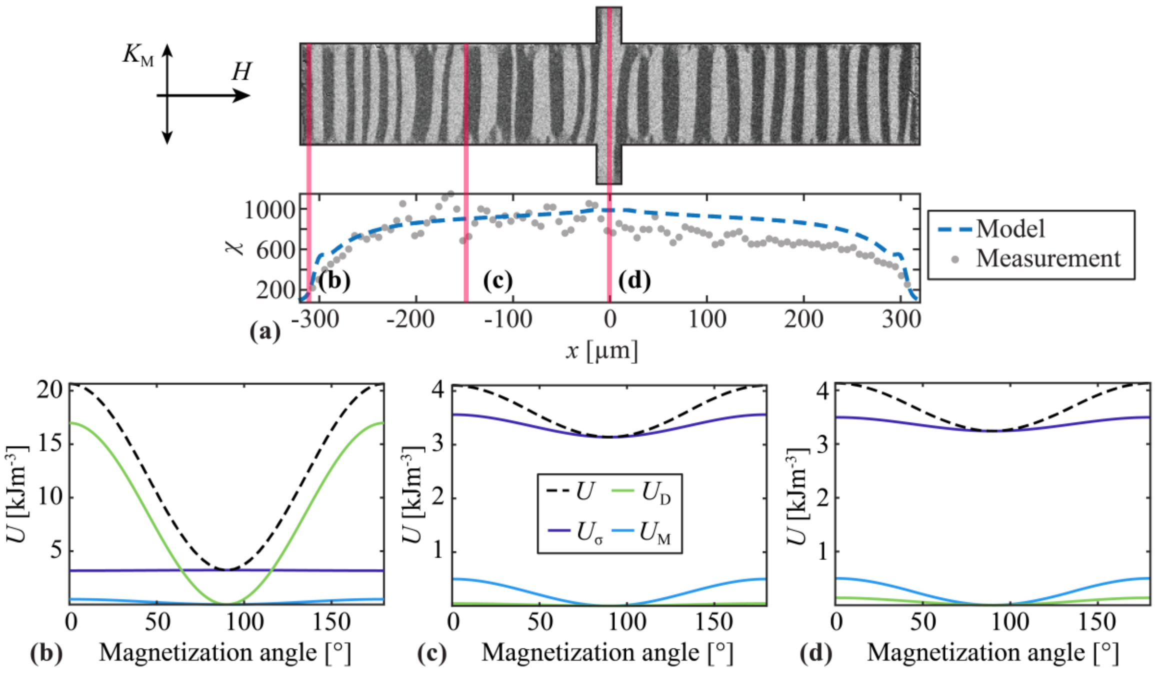

Figure S4. Comparison of various energy contributions to the total energy of the macrospin at three different positions on the x-axis. a) MOKE image of the magnetic domains of the sensor after demagnetizing along the x-axis and spatial distribution of the differential magnetic susceptibility χ estimated from the measurements and compared with simulations (identical to Figure 3a-b in the main text). The sketch on the left indicated the orientation of the applied magnetic field H and the uniaxial magnetization-induced anisotropy K_M. b-d) Dependency of the energy density of the macrospin (Eq. S1) on the magnetization angle for x = -315 µm (b), x = -150 µm (c), x = 0 µm (d).

S5. Magnetic Properties of Different Sensor Geometries

In this study, we investigated twelve single sensors with varying geometries, i.e., in-plane dimensions and anchor width, and two sensor arrays with identical ten and fourteen parallel-connected sensors from the same chip while focusing on one sensor (Sensor ID1) in detail. Below, magnetic domain images in a demagnetized state (Figure S5) and magnetization curves (Figure S6) of the single sensors (ID2-12) can be found. Detailed magnetic properties of ID1 are given in the main text.

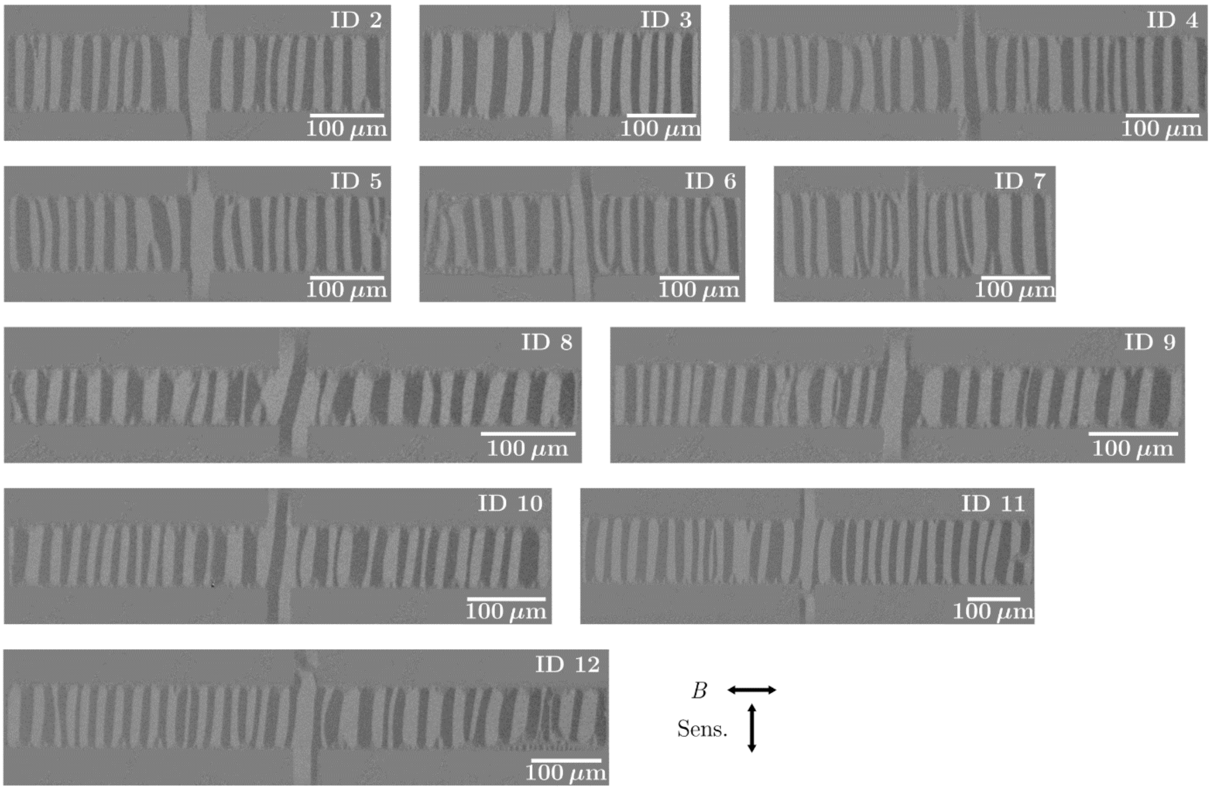

Figure S5. Magnetic domain images of the investigated sensors (ID2-12) after demagnetizing the sensor along their long axes. Magneto-optical sensitivity is aligned perpendicular to the demagnetizing field.

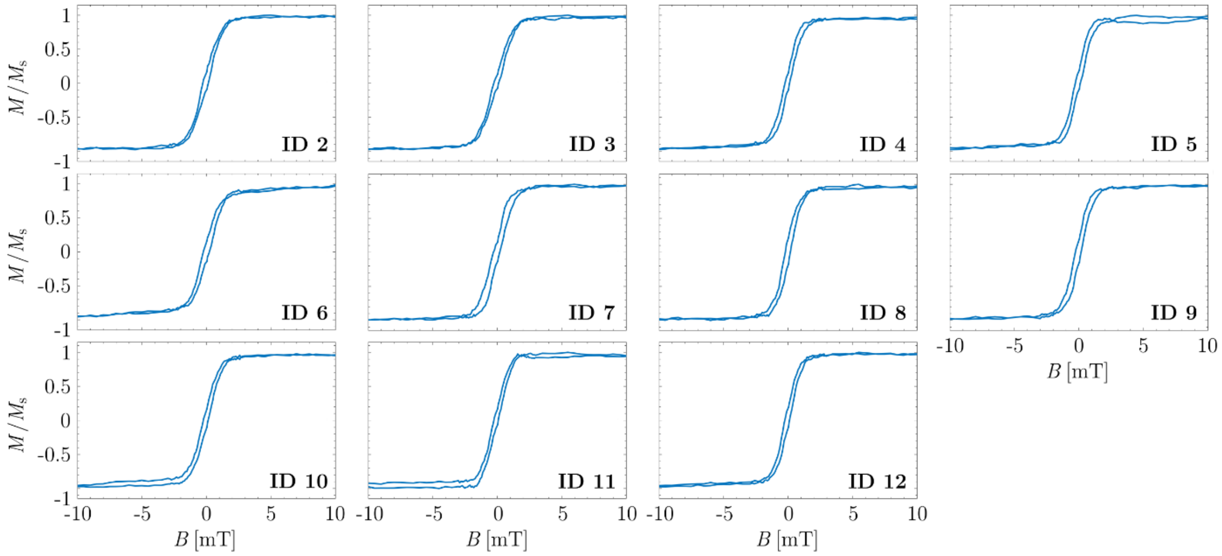

Figure S6. Magnetization curves of the investigated sensors (ID2-12) measured along their long axes.

S6. Nonlinearity

We measured the admittance magnitude of the sensor ID1 at its magnetic working point and different excitation voltage amplitudes and determined the electrical sensitivity. Figure S7a shows that the resonance peak in the admittance curve shifts toward smaller frequencies with increasing excitation voltage amplitude u_{ex}, with reduced electrical sensitivity, which becomes significantly visible above 100 mV. To understand the origin of this nonlinearity, admittance magnitude measurements were also done at the magnetic saturation (Figure S7b). In contrast to measurements at the magnetic working point, admittance magnitude and electrical sensitivities are independent of the excitation voltage amplitude. It shows that nonlinearity results from a magnetostrictive origin. This study used an excitation voltage amplitude of 50 mV for all measurements since the sensors are still in a linear regime with sufficient signal amplitude.

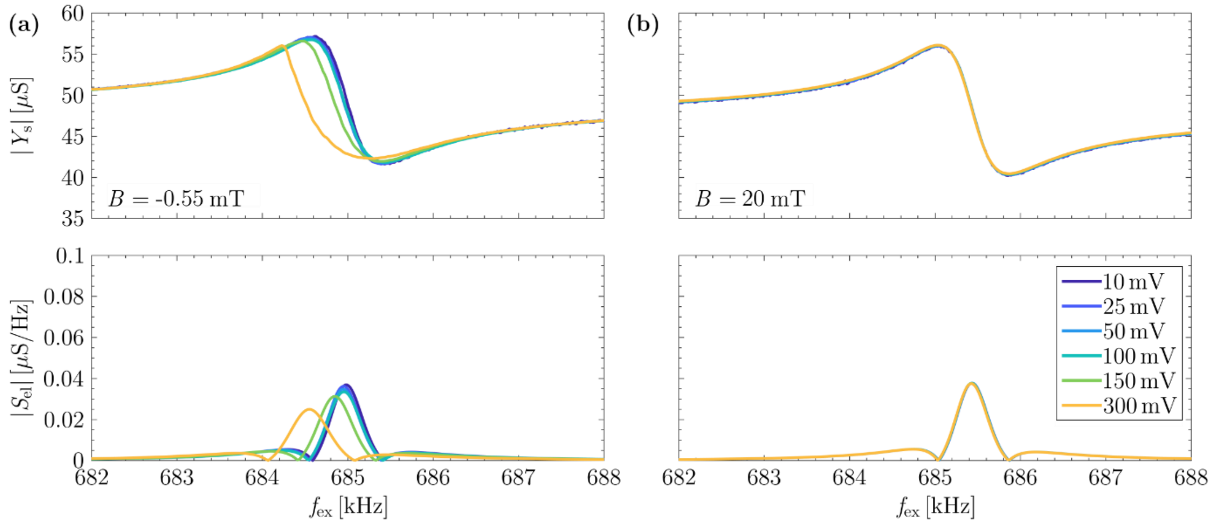

Figure S7. Nonlinearity-excitation voltage amplitude relationship. (**a**) Admittance magnitude $|Y_s|$ and magnitude of electrical sensitivity $|S_{\mathrm{el}}|$ of sensor ID1 for RM3 at different excitation voltage amplitudes and magnetic bias field of $B = -0.55$ mT and (**b**) $B = 20$ mT.

S7. Stress Components in RM1-RM4

The stress magnitudes of the four resonance mode shapes are shown in Figure S8. In RM3, a significant contribution of the shear stress component σ_{12} is apparent.

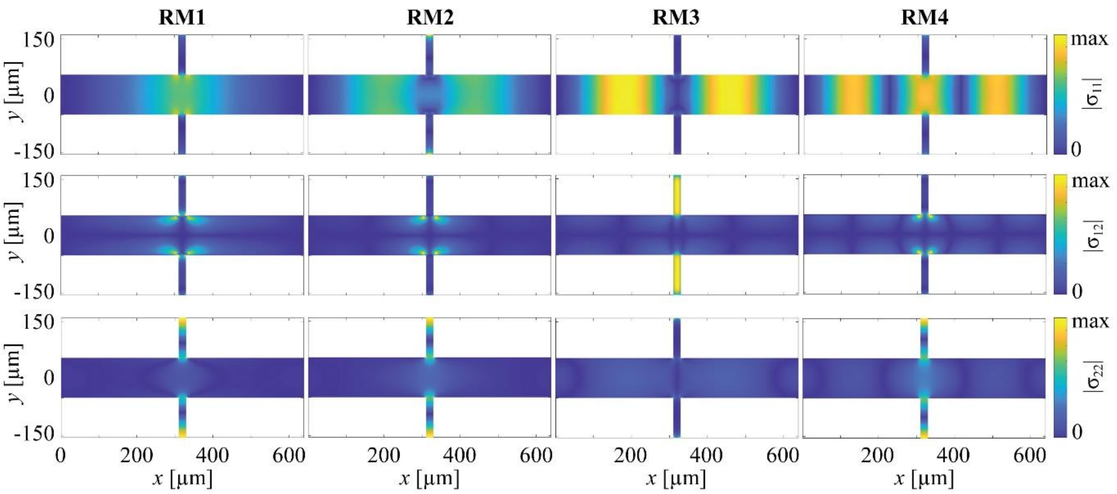

Figure S8. Stress distribution. Magnitudes of the three stress components σ_{11}, σ_{12}, σ_{22} in the four resonance modes RM1-4 in the center of the magnetic layer.

S8. Sensor Characteristics

Resonance frequencies, sensitivities, and quality factors for RM1-4 of the single sensors, except for RM1 of Sensor ID3 and ID11 (due to noise) and RM1,2 of ID4 (due to a broken sensor), are determined. The geometries and the resonance frequencies f_r of the investigated single sensors are given in Table S1. Relative magnetic, relative electrical and total amplitude sensitivities are shown in Table S2. Quality factors at zero bias field can be found in Table S3. Table entries that could not be determined due to noise (RM1 of ID3 and ID11) and a broken resonator (RM1 and 2 of ID4) marked with "X".

Sensor ID	In-plane dimensions [µm ×µm]	Anchor width [µm]	f_r [kHz] RM1	RM2	RM3	RM4
1	640 × 105	25	125.7	365.9	685.2	1301.8
2	510 × 105	25	171	479.7	1072.1	1985
3	400 × 125	25	X	676.1	1734	2692
4	640 × 105	30	X	X	690.8	1298.6
5	510 × 105	30	176.9	490.8	1080.5	1985.4
6	400 × 100	30	235.1	699.5	1747.3	2792.7
7	400 × 125	30	210	681.6	1741	2643.4
8	600 × 60	30	157.1	488.9	798.5	1448.9
9	640 × 70	30	137.9	437.2	700.2	1295.3
10	690 × 80	30	114.4	340.3	598.3	1106.7
11	850 × 125	30	X	218.8	390.8	745.3
12	850 × 90	35	79.8	276	398.4	757.8

Table S1. Sensors' parameters. In-plane dimensions, anchor widths, and measured resonance frequencies f_r of the first four resonance modes RM1-4 at zero magnetic bias field.

Sensor ID	$S_{\mathrm{m,r}}$ [T^{-1}]				$S_{\mathrm{el,r}}$ [mS]				S_{am} [µS mT^{-1}]			
	RM1	RM2	RM3	RM4	RM1	RM2	RM3	RM4	RM1	RM2	RM3	RM4
1	8.9	2.7	6.2	4.8	0.8	4.0	19.5	2.8	7	11	121	14
2	7.4	3.0	6.9	4.3	0.4	10.3	12.5	2.0	3	31	86	9
3	X	6.4	5.2	2.4	X	4.7	4.9	1.2	X	30	25	3
4	X	X	5.6	4.3	X	X	20.8	2.2	X	X	116	9
5	7.6	2.9	5.5	4.9	0.6	9.0	14.7	2.1	5	25	81	10
6	3.7	2.6	3.4	1.1	0.2	8.9	6.5	3.4	1	23	22	4
7	4.6	3.6	3.8	1.5	0.0	7.6	5.8	6.3	0	27	22	9
8	4.9	1.7	4.1	4.1	0.9	2.4	11.9	2.0	4	4	49	8
9	7.5	2.0	6.4	4.2	0.7	3.8	14.9	4.5	5	8	95	19
10	5.8	2.3	6.2	5.0	1.1	3.9	13.7	4.2	6	9	85	21
11	X	3.1	8.2	6.1	X	2.5	12.7	4.2	X	8	104	26
12	9.9	3.1	8.2	5.5	0.7	2.6	12.6	2.8	7	8	103	15

Table S2. Sensitivities of the investigated sensors. Relative magnetic $S_{m,r}$, relative electrical $S_{el,r}$, and total amplitude sensitivities S_{am} at magnetic working points for RM1-RM4.

Q	Sensor ID											
	1	2	3	4	5	6	7	8	9	10	11	12
RM1	609	525	X	X	608	640	422	690	551	621	X	490
RM2	687	828	572	X	732	681	604	683	744	670	459	594
RM3	814	579	319	764	596	355	264	945	920	838	754	789
RM4	394	264	342	363	265	515	517	512	689	624	508	605

Table S3. Quality factors of the investigated sensors at zero magnetic bias field for RM1-RM4.

References

[1] B. Spetzler, C. Kirchhof, E. Quandt, J. McCord, F. Faupel, *Phys. Rev. Appl.* **2019**, *12*, 64036.

[2] E. C. Stoner, E. P. Wohlfarth, *IEEE Trans. on Magn.* **1991**, *27*, 3475.

[3] J. Lou, R. E. Insignares, Z. Cai, K. S. Ziemer, M. Liu, N. X. Sun, *APL* **2007**, *91*.

[4] A. Ludwig, E. Quandt, *IEEE Trans. on Magn.* **2002**, *38*, 2829.
